# Advancing Ego Development in Adulthood Through Study of the Enneagram System of Personality

**DOI:** 10.1007/s10804-018-9289-x

**Published:** 2018-01-30

**Authors:** David Daniels, Terry Saracino, Meghan Fraley, Jennifer Christian, Seth Pardo

**Affiliations:** 1 Stanford Medical School, Psychiatry and Behavioral Sciences, Palo Alto, CA USA; 2 The Narrative Enneagram, Boulder, CO USA; 3 Denver, CO 80210 USA; 4 Department of Social Sciences, Skyline College, San Bruno, CA USA; 5 Mountain View, CA 94041 USA; 6 Sofia University, Palo Alto, CA USA; 7 San Francisco, CA 94118 USA; 8 California School of Professional Psychology, Alliant International University, San Francisco, CA USA; 9 San Francisco, CA 94133 USA

**Keywords:** Enneagram, Ego development, Personality, Postconventional development, Adulthood

## Abstract

A rapidly growing number of working professionals, academic communities, and businesses have applied the Enneagram personality system of nine types to enhance psychological growth in their personal and professional lives. However, there are no existing studies that measure the effects of the application of Enneagram training programs to promote ego development. This study examined if ego development took place among individuals enrolled in Enneagram training programs in the Narrative Tradition. Two groups of participants (*N* = 122) were assessed using the Washington University Sentence Completion Test (WUSCT) at baseline (pretest) before the training began and then again 18 months later (posttest); one of the groups participated in Enneagram Intensive training programs (*n* = 73) and the other group participated in introductory Enneagram trainings (*n* = 49). Data revealed an advancement in ego development among some of the participants who received at least 40–50 h of training. The findings suggest that Enneagram Intensive trainings may be beneficial for promoting psychological growth and ego development. Clinical applications and future research directions are discussed.

## Introduction

The Enneagram personality system is rapidly expanding as a tool for working with personality structure and personal development in a diverse array of contexts including businesses, governmental agencies, education, and the human services fields (Bland 2010). Training programs use this system to help facilitate greater psychological health, interpersonal effectiveness, and ego development (Daniels 2012; Gallant 2005; Lapid-Bogda 2010). Despite widespread application for the purpose of adult development, there is a lack of rigorous scientific research on the application of the system (Fitzsimons and Killen 2013; Sutton 2012). Loevinger’s stage theory of ego development (Loevinger 1976) provides a framework through which the impact of Enneagram training on ego development may be explored. The aim of this study was to identify the potential of promoting ego development in adulthood through training with a foundation in the Enneagram personality system.

### Loevinger’s Theory of Ego Development

The Loevinger theory of ego development is one of the most active theoretical traditions within personality psychology (Kurtz and Tiegreen 2005; Loevinger 1976). Loevinger’s ego developmental approach dovetails with the Enneagram personality system. Both are holistic systems of personality and development. The systems share the premise that ego is a schema through which people make meaning and can develop higher levels of functioning with an expanded worldview and greater empathy (Daniels and Price 2009; Kurtz and Tiegreen 2005; Manners et al. 2004). However, research on ego development using Loevinger’s model has demonstrated that the majority of adults in the general population do not reach the highest levels of functioning. Instead, there is stabilization at conventional levels of development in adulthood, demonstrating that only about 10% of adults reach postconventional levels (Cook-Greuter 1999, 2011; Manners et al. 2004).

The Loevinger model of ego development (1976) is comprehensive; addressing character development, cognitive complexity, interpersonal style, and conscious preoccupations (Chandler et al. 2005; Loevinger 1976). The model draws from and expands upon the work of other developmental theorists (Brown 2012; Cook-Greuter 1990; Kohlberg 1976; Lambie 2007; Pfaffenberger 2007; Piaget 1977). Loevinger’s theory delineates nine hierarchical stages of ego development progressing from infancy through adulthood. According to Loevinger’s definition of stage, new stages are qualitatively different from the preceding one. This theory of stage transition rests upon Piaget’s paradigm of ego development in which there are dynamic shifts between stages that are qualitatively distinct stages (Cook-Greuter 1999; Hy and Loevinger 1996; Pfaffenberger and Marko 2011) (Table 1). Development is often initiated by awareness of persistent inconsistencies that are not compatible with one’s current paradigm. To resolve these inconsistencies, a reorganization of one’s operating paradigm leads to development and greater integration. At each progressive stage, the individual exemplifies ways of meaning-making that were not available at earlier stages with increases in awareness, autonomy, self-regulation, and complexity of thinking (Lambie 2007; Manners et al. 2004).

**Table 1:** Loevinger developmental model

Developmental category	Adult population	Stage	Developmental stage name
Preconventional	10%	2	Impulsive
		3	Self-protective
Conventional	80%	4	Conformist
		5	Self-aware
		6	Conscientious
Postconventional	10%	7	Individualist
		8	Autonomous
		9	Construct aware
		10	*Unitive* ^a^

^a^Cook-Greuter (1999)

The conceptual soundness of the Loevinger developmental theory has been supported by rigorous research (Cohn and Westenberg 2004; Manners and Durkin 2001). For the purposes of research and assessment, Loevinger developed a projective assessment instrument for measuring these stages: the Washington University Sentence Completion Test (WUSCT). The Loevinger model and assessment tool have become a standard for ego development research, and to date they have been the basis for hundreds of investigations of adult ego development (Pfaffenberger and Marko 2011).

Cook-Greuter (1994) classified the nine distinct stages of Loevinger’s model into three hierarchical categories: preconventional, conventional, and postconventional levels (see Table 1). The preconventional stages are estimated to constitute 10% of the adult population and are described as Symbiotic, Stage 1; Impulsive, Stage 2; and Self-protective, Stage 3. These stages are normal in childhood, but if not progressed beyond, may be associated with psychopathology in adulthood (Pfaffenberger and Marko 2011). The conventional stages, estimated to constitute about 80% of the adult population, are described as Conformist, Stage 4; Self-Aware, Stage 5; and Conscientious, Stage 6. These levels represent a shift typically occurring after age 12 when the individual becomes capable of taking in another’s perspective and develops a clearly separated identity and self-contained integrated system (Akrivou 2008). The postconventional stages are estimated to constitute about 10% of the US adult population and are described as Individualistic, Stage 7; Autonomous, Stage 8; and Construct-aware, Stage 9. In postconventional stages, the individual has greater differentiation of the self and demonstrates greater psychological integration, self-actualization, wisdom, and access to intuition (Cook-Greuter 2000). According to Cook-Greuter (1999) there is a 10th stage of development, Unitive, where individuals can sustain an ongoing openness to experience where transpersonal experiences are integrated into a fluid state of being.

### The Enneagram and Ego Development

Loevinger’s (1976) ego development model provides a useful framework for measuring ego development in the Enneagram system. Developmental trajectories are then available for each Enneagram type to follow in order to achieve higher levels of ego development. The shifts in worldview toward higher levels of development in the Enneagram are consistent with the achievement of higher developmental levels within Loevinger’s model (Bland 2010; Loevinger 1976; Cook-Greuter 2002; Maslow 1964). Higher levels are marked by increased awareness, empathy, and less identification with ego (Cook-Greuter 2002; Jaxon-Bear 2012; Loevinger 1976). Literature indicates that mental health and ego development are interrelated, with positive correlations between development and dispositional optimism and self-esteem, and negative correlations with affective distress, depression, and anxiety (Blumentritt 2011; King et al. 2000; Noam et al. 2006). Ultimately, development leads to self-actualization and self-transcendence/unity as described in the frameworks of both Loevinger (1976) and Maslow (1976).

### The Enneagram of Nine Types

The Enneagram is a personality system of nine distinct type-specific paths for development across the lifespan (Bland 2010; Daniels and Price 2009; Sutton 2012). Many researchers have highlighted the Enneagram system’s parallels to that of other contemporary psychological theories such as humanistic, psychodynamic, Jungian, cognitive–behavioral therapy and developmental psychologies and the field of neuroscience (Beesing et al. 1984; Daniels and Price 2009; Naranjo 1990, 1994; Palmer 1988, 1995; Riso and Hudson 1999, 2005; Siegel 2004, 2010; Tolk 2006; Wagner 2008). The majority of books and articles exploring the application of the Enneagram system are in the realm of personal development and growth. More recently, the Enneagram has expanded to the area of organizational development. In the work setting it has been used as a tool for team building and facilitating more harmonious workplaces (Bland 2010; Colina 1998; Kale and Shrivastava 2003; Ormond 2007).

Each Enneagram type is distinct with its own particular strengths, core beliefs, limitations, and approach to relationships (Sutton 2012). As a comprehensive personality system, the Enneagram types portray nine fundamental patterns (Table 2). Within the Enneagram system these interwoven patterns are described in terms of the type’s distinct perceptual filters that form the Basic Proposition (Daniels and Price 2009). The Basic Proposition represents the intersection of: (a) core beliefs; (b) adaptive strategies; (c) habits of attention; and (d) driving emotions. Within the development of each type, the full range of human potential can be achieved through the course of a lifespan (Bland 2010).

**Table 2:** Brief descriptions of the nine patterns (Reprinted with permission from Daniels and Price 2009)

**Type one: The perfectionist** believes you must be good and right to be loved, secure, and worthy. Consequently, perfectionists are conscientious, responsible, improvement-oriented, and self-controlled, but also can be critical, resentful, and judgmental.
**Type two: The giver** believes you must give fully to others to be loved, secure, and worthy. Consequently, givers are caring, helpful, supportive, and relationship-oriented, but also can be prideful, overly intrusive, and demanding.
**Type three: The performer** believes you must accomplish and succeed to be loved, secure, and, worthy. Consequently, performers are industrious, fast-paced, goal focused, and efficiency-oriented, but also can be inattentive to feelings, impatient, and image-driven.
**Type four: The romantic** believes you must obtain the longed for ideal relationship or situation to be loved, secure, and worthy. Consequently, romantics are idealistic, deeply feeling, empathetic, authentic to self, but also dramatic, moody, and sometimes self-absorbed.
**Type five: The observer** believes you must protect yourself from a world that demands too much and gives too little to be loved, secure, and worthy. Consequently, observers are self-sufficient seeking, non-demanding, analytic/thoughtful, and unobtrusive, but also can be withholding, detached, and overly private.
**Type six: The loyal skeptic** believes you must gain protection and certainty in a hazardous world you just can’t trust in order to be loved, secure, and worthy. Consequently, loyal skeptics are themselves trustworthy, inquisitive, good friends, and questioning, but also can be overly doubtful, accusatory, and fearful.
**Type seven: The epicure** believes you must keep life up and open to assure a good life and be loved, secure, and worthy. Consequently, epicures are optimistic, upbeat, possibility and pleasure seeking, and adventurous, but also can be pain-avoidant, uncommitted, and self-serving.
**Type eight: The protector** believes you must be strong and powerful to assure protection, love, and worthiness in a tough world. Consequently, protectors are justice seeking, direct, strong, and action-oriented, but also overly impactful, excessive, and sometimes impulsive.
**Type nine: The mediator** believes that to be loved, secure, and worthy you must blend in and go along to get along. Consequently, mediators are harmony seeking, comfortable, and steady, but also self-forgetting, conflict avoidant and sometimes stubborn.

The majority of research on the Enneagram has focused on confirmation of the construct validity of the types and predictive ability of assessment instruments (Bland 2010; Sutton 2012). Research has demonstrated that there is empirical validity to the system and predictive ability of instruments (Daniels and Price 2009; Wagner 1983). Positive correlations to other personality scales have been demonstrated, including the Occupational Personality Questionnaire (OPQ), the Big Five traits (Bartram and Brown 2005), and subsequent projective tests (Sutton 2012). Consistent with Enneagram theory, research has shown that an individual’s specific Enneagram type remains stable over time, with an average stability of 85% using the Million Illinois Self-Report Inventory Scales and Myers–Briggs Type Indicator (Wagner 1983).

Research on the application of the Enneagram has shown its potential to effectively promote development in a number of areas. In a business setting, one study demonstrated an increase in team effectiveness after training in the Enneagram (Ormond 2007). Sutton et al. (2013) looked at the potential of Enneagram training to promote personal development and improved attitude toward work. They found that participants who took a 4-h introductory Enneagram workshop at their workplace reported subsequent increased appreciation of diversity, self-confidence, and enhanced communication skills. However, Sutton and colleagues did not find an increase in self-awareness among the participants. Other studies have demonstrated that the Enneagram can facilitate gains in psychological health including greater self-understanding, self-esteem, interpersonal skills, and the reduction of anxiety (Azimipour 2009; Rasta et al. 2012).

There remains a paucity of empirical research on the application of the Enneagram, particularly in the realm of personal development (Bland 2010; Sutton 2012). In particular, a limitation of past studies has been the short duration of trainings lasting from a few hours up to a day. To really illuminate the potential of the system to harness greater self-awareness, studies with trainings of a longer duration are needed. While short trainings may be helpful in some respects, to see the extent of the potential for training to promote ego development and self-awareness, participants likely need more time to engage with the material in an experiential and personally meaningful way. Furthermore, research with longer follow-up periods is needed to assess sustained changes.

### How the Enneagram Facilitates Personal Development

The Enneagram personality system offers a comprehensive guide to type-specific personalities and their associated strengths, limitations, and core beliefs. The system serves as a roadmap toward personal development throughout the lifespan with unique aspects linked to the developmental objectives for each type. Studying the Enneagram type structure fosters deepening self-awareness of the individual’s mental, emotional, and somatic perceptual filters (Saracino 2013; Tolk 2006). As previously unconscious patterns and beliefs become conscious, the individual re-examines and re-evaluates previously held worldviews leading to healthier stages of psychological and ego development. While there are distinct ego structures of each type, common to the developmental path of all types is an increase in awareness and empathy and less identification with ego.

### The Narrative Tradition Enneagram Training Approach

The Enneagram training in the Narrative Tradition integrates the content of the Enneagram with a learning process that is designed to support personal development. The training comprised four components that interweave content and process: type panels, didactic learning, experiential exploration, and reflective practices (Daniels 2012; Saracino 2013). At its core, the training invites self-exploration and offers tools for how to bring the lessons learned into behavioral practice (Saracino 2013).

The type panels are the foundation of the Narrative Tradition approach. In a type panel, a trained facilitator interviews a group of representatives of a particular Enneagram type (Saracino 2013). The panel is often preceded by a breath practice used to cultivate a state of compassion, mindfulness, and self-observation for both panelists and the audience. The facilitator prompts the panelists to share their experiences. The shared patterns of the type structure are revealed through their verbal and nonverbal communication. The interactive sharing of personal stories cultivates an atmosphere of openness and an experience of shared humanity that is the groundwork for learning.

Through integration of the theoretical framework of the Enneagram, self-reflective processes, and experiential exploration, participants may translate personality insights into changes in ego functioning. The reflective practices are designed to allow participants to obtain greater understanding of their own perceptual schemas, mental preoccupations, and worldview (Siegel 2007, 2010). Experiential exploration then allows participants to recognize the limitations of their current worldview in effectively navigating their environment (Marko 2011). The Enneagram model provides a type-specific framework for the individual to develop and adopt a more integrative complex worldview. The individual thus may progress to a higher level of ego development through a process reflective of Piaget’s (1977) concept of assimilation and accommodation. The work also involves integration of all three centers of intelligence: mental, emotional, and somatic.

### Interventions to Facilitate Ego Development

Ego development research in recent years has explored whether it is possible to promote ego development through interventions in adulthood. Historically, a prominent view within personality and developmental psychology is that personality stabilizes during adulthood (Caspi and Bem 1990; McCrae and Costa 1980; Manners et al. 2004). Research has demonstrated that the majority of adults’ development stabilizes at the self-aware ego stage at 80%, within the conventional strata of development (Cohn 1998; Loevinger et al. 1985). However, in recent years research has demonstrated that personality can change in adulthood, and it is possible to advance ego stages in adulthood (Alexander et al. 1990; Hurt 1990; Lasker and Strodtbeck 1975; MacPhail 1989; Staudinger and Kunzmann 2005; White 1985).

In recent years, postconventional stages of personality development have become a quickly growing area of interest (Pfaffenberger and Marko 2011). In the empirical research, the potential to intentionally promote postconventional development is largely unexplored. Thus far, Chandler et al. (2005) conducted a longitudinal experimental study that explored the impact of transcendental meditation (TM) on ego development, and examined potential development from conventional to postconventional stages. At 10-year follow-up, they found that 38% of the intervention group that practiced TM achieved a postconventional stage as compared to 1% of the control subjects. As a result, they suggested the practice of systematically transcending representational thought and experiencing pure consciousness is a necessary component to achieving postconventional stages.

Despite the possibility of ego development in adulthood, little research exists on interventions that promote ego stage advancement (Alexander et al. 1990; Chandler et al. 2005; Hurt 1990; MacPhail 1989; Manners et al. 2004; White 1985). The research that has been conducted has indicated that interventions can be effective. Manners et al. (2004) designed a training program that integrated four components of experiences they hypothesized as essential for promoting ego development: experiences that are (a) structurally disequilibrating, (b) personally salient, (c) interpersonal, and (d) emotionally engaging. They found that the intervention groups demonstrated increased levels of ego development in contrast to the control group. As a result, Manners and colleagues concluded that it is possible to construct interventions to promote ego stage advancement and asserted that these interventions are able to do so in so far as they are disequilibrating, salient, interpersonal, and emotional.

The Enneagram training program in this study is both consistent theoretically with Loevinger’s model, and consistent with the intervention components identified as promoting ego development (Manners et al. 2004). We hypothesized that training in the Enneagram can promote ego development in adulthood. The study design accounted for some limitations in previous research on both the Enneagram and Ego Development. The training program was of sufficient duration to allow the researchers to assess the impact of the Enneagram training program, and posttest was at 18 months follow-up in order to evaluate stage change that was sustained.

### The Present Study

This study examined the impact of training using the Enneagram system of nine personality styles as a framework that could promote ego development. We explored whether or not individuals in the Enneagram Intensive Training Groups would demonstrate a significant increase of ego development following participation in these trainings. We also explored whether individuals in introductory level Enneagram workshops demonstrated any changes in ego development. The authors expect that all participants will benefit to some degree in ego development following the trainings, regardless of whether the trainings are intensive or introductory. The authors also expect that participants in the Intensive Training Groups will report significantly more ego development than participants in the Comparison Group; that is, participants with more intensive Enneagram training will demonstrate significantly more ego development than participants with introductory Enneagram training.

## Method

### Participants

Participants learned about the Enneagram training courses through multiple sources: word-of-mouth, attendees from prior Enneagram trainings, flyers on extensive Enneagram Worldwide mailing lists, attendees at Enneagram conferences, and Narrative Tradition program graduates. Participants arrived at the training knowing their own type. If a participant was unsure about their Enneagram type, instructors encouraged them to reflect during the training to distinguish which type most resonated with them. At the beginning of each training, individuals were asked whether they wanted to be included in the study.

A total of 162 people agreed to participate in the ego development study. Complete data are provided here on 122 participants who completed both portions of the ego development measure. Data from 26 enrollees who did not complete the study were not included in the analyses. An additional 14 participants from an introductory training group participated in an unscheduled extra training session before their posttest, so their data were not included in this analysis as the unanticipated extra training hours could have influenced their posttest scores in a manner not in alignment with the intended study design. Enneagram Intensive trainings ranged from 45 to 50 training hours, and the Enneagram Introductory trainings ranged from 10 to 20 h.

#### Sample

A final sample resulted in 122 participants (*n* = 73 Intensive; *n* = 49 Introductory). A summary of participant demographic characteristics is shown in Table 3.

**Table 3:** Sample demographic information (*N* = 122)

Variable	Total sample *N* (%)	Intensive groups *N* (%)	Introductory groups *N* (%)
Sex			
Female	100 (82)	59 (48)	41 (34)
Male	22 (18)	14 (11)	8 (7)
Age (years)			
Under 30	5 (4)	2 (2)	3 (3)
30–39	3 (3)	1 (1)	2 (2)
40–49	28 (23)	16 (13)	12 (10)
50–59	53 (43)	35 (29)	18 (15)
60+	33 (27)	19 (16)	14 (11)

Sample participants were predominantly female (*n* = 100; 82%), and comprised mostly middle-aged adults (*n* = 86; 70% age 50+). Analysis of group demographics was conducted to assess for any important differences between samples. Chi-square analysis indicated no significant differences between levels of ego development in the two groups at pretest. Chi-square analysis showed no significant differences between the groups in regard to gender and age.

### Materials

#### Washington University Sentence Completion Test (WUSCT) [Updated by Cook-Greuter]

Jane Loevinger’s Washington University Sentence Completion Test (WUSCT) (Loevinger and Wessler 1970; Hy and Loevinger 1996) updated by Cook-Greuter (1999) was used to measure levels of ego development. Cook-Greuter’s updated version included Construct Aware (Stage 9) and Unitive (Stage 10). To avoid measurement error that would result from repeat testing in a within-subjects design, alternate short forms of the WUSCT updated by Cook-Greuter (1999) were given at pretest and posttest (Redmore and Waldman 1975). Alternate forms derived from WUSCT Form 81 consisted of 18-items each, the first half given at pretest and the second half given at posttest (Hy and Loevinger 1996). Correlation of the first and second halves was very high (*r* = .96) with a high Cronbach’s coefficient (*α* = 0.91) (Novy and Frances 1992). Participants were prompted to complete sentences from basic prompts. Items included: “What gets me into trouble is…” and “I feel sorry…” The WUSCT scale score assesses the test taker’s level of development within Loevinger’s ego development model as was shown in Table 1.

Several studies indicate that the WUSCT is a reliable and valid psychometric measurement of ego development (Cook-Grueter and Soulen 2007; Lilienfeld et al. 2000; Manners and Durkin 2001; Noam et al. 2006; Pfaffenberger 2011). There is strong inter-rater reliability of Cook-Greuter’s modifications to Loevinger’s model (*r* = .95). It is important to note that the WUSCT may be significantly affected by the motivation of the test taker (Redmore 1983; Kitchener et al. 1984; Loevinger et al. 1985). For example, with no motivation or distraction, there can be an artificial decrease in scores (Drewes and Westenberg 2001). However, one cannot score at a higher stage than they have in reality achieved. In other words, a participant can intentionally score lower, but people are not good at artificially increasing their score. Given the well-documented downward drift in scores at posttest, the primary purpose of the instrument in this study was to identify participants who were able to advance stages.

### Procedure

Eight Enneagram training courses were offered between 2005 and 2007, five of which were intensive courses and three of which were introductory courses. The Enneagram Intensive trainings offered 45–50 training hours, and the Introductory Enneagram trainings offered between 10 and 20 training hours. A baseline assessment (pretest) occurred before the training began and a follow-up assessment (posttest) occurred 18 months after the baseline assessment.

The first 18 stems of WUSCT Form 81 were administered at the beginning of each of the trainings. 18 months after administration of the pretest, the last 18 stems of the WUSCT were mailed to each study participant for a follow-up assessment. A self-addressed stamp envelope was provided for participants who were asked to complete the form within 2 weeks and return it to the provided return address by mail. There was an 84% return rate of the mailed WUSCT forms (*n* = 136 returned of 162 participants). After the 14 forms were removed from those who participated in the unscheduled extra training session, the final sample consisted of 122 participants.

#### Enneagram Intensive Trainings

The Enneagram Intensive Courses ran at various times and various locations between 2005 and 2006, with mostly the same instructors. Two trainings each totaling approximately 45 h took place in Asheville, North Carolina, between 2005 and 2006, and both trainings were co-facilitated by the same core faculty (DD and TS). Three trainings each totaling approximately 50 h took place in 2006. Two of the three trainings took place in Menlo Park, California (one in January and one in August), and a third training took place in Scottsdale, AZ. Core faculty DD co-facilitated all three of these trainings. Core faculty TS co-facilitated the two Menlo Park trainings, and another core faculty PO co-facilitated both Menlo Park (January, 2006) and Scottsdale (2006).

#### Introductory Enneagram Trainings

The three Enneagram Introductory Courses were offered between 2006 and 2007 and offered between 6 and 20 training hours. The first Introductory course took place on a single day in Berkeley, California, with core faculty PO and instructor PC; this 1-day training lasted approximately 6 h. The second Introductory course took place over a single weekend in Minnesota in 2007 with core faculty DD and TS and lasted approximately 13 h in total. The third Introductory course took place weekly over 10 weeks in Palo Alto, California, between March and May, 2006 with four instructors JK, SS, LH, and NV, and in total offered approximately 20 training hours.

#### The Intervention

The Enneagram trainings in this study used the Narrative Tradition method. The key feature of this method is teaching the Enneagram by interviewing representatives of the nine types on what is called a type panel. Enneagram type panels are a teaching method that promotes open exploration of each personality type. A type panel comprised individuals who represent the type being featured by sharing their strengths and limitations of self-observations, mental preoccupations, and strengths and weaknesses of the type (Palmer 1995). Enneagram type panels comprised people currently enrolled in the Enneagram course and if needed attendees of previous workshops that identify with one of the nine types of the Enneagram. Type panels help educate current course enrollees about the basic themes of the nine different Enneagram types, and through instructor-facilitated interviews with the panelists, also help promote greater understanding and insight into the lived experiences of the nine different Enneagram types.

Didactic learning included lectures, and question and answer segments. The didactics in the study focused on the theoretical frameworks of each type including: (a) differences in mental preoccupations; (b) process and tasks of personal development; (c) core beliefs; (d) driving emotions; (e) awareness of patterns and reactivity; and (f) strengths and challenges of each type.

Reflection exercises encourage personal inquiry through worksheets, small groups, and dyad exercises. These exercises promote self-awareness, reflection on relationship styles, identification of current developmental tasks, and paths to personal growth. The fourth learning modality, experiential exploration, includes: (a) guided visualizations; (b) mindful awareness practices; and (c) bodywork and movement. These experiential explorations promote increased awareness of emotional and mental preoccupations of the nine types and their corresponding bodily sensations.

All instructors in the study were certified Enneagram teachers in the Narrative Tradition. The Enneagram Intensive Training Courses used four primary learning modalities: (1) Enneagram type panels, (2) didactic learning, (3) reflection exercises, and (4) experiential exploration. Enneagram Intensive Trainings instructors followed an outline entitled The Enneagram Intensive®: Integrating Psychological Life and Spirit.

The three Introductory training courses incorporated primary Enneagram learning modalities. The Berkeley course incorporated informal Enneagram type panels and didactic learning. The Minnesota course used all four of the primary learning modalities. The Palo Alto course used Enneagram type panels, didactic learning, and experiential exploration such as guided visualization.

### Data Analysis

Questionnaires were typed by a research assistant and sent to a Cook-Greuter-certified SCT scorer (Hy and Loevinger 1996) via post mail. Code numbers were used so that the rater was blind to the nature of the sample, the dates of the sample, and the training group type. The scorer followed the training by Susan Cook-Greuter, using the original Loevinger manual, except for stems not in the manual (Cook-Greuter 1999). Scored responses were sent back to the researchers in an Excel spread sheet which was then used for data analysis. To ensure reliability of the scoring for results in which participants went up or down two or more stages, a sample of those studies was also scored by Cook-Greuter herself.

## Results

Table 4 summarizes the descriptive ego development scores by site for both pre-training and post-training time points.

**Table 4:** Ego development scores at pretest and posttest

Training site	*N*	Pretest		Posttest		*t*	*df*
		*Mean*	*SD*	*Mean*	*SD*		
Intensive							
Asheville (2005)	23	6.3	1.1	7.1^a,b^	0.9	− 3.36**	22
Asheville (2006)	11	6.5	1.3	6.5^a^	1.1	− 0.29	10
Vallombrosa (Jan 2006)	14	6.1	1.2	6.0^b^	1.1	0.46	13
Vallombrosa (Aug 2006)	11	6.4	0.9	6.5^a^	1.1	− 0.21	10
Scottsdale (2006)	14	6.4	1.1	6.6^a^	1.3	− 0.44	13
Introductory							
Minnesota (2007)	34	6.5	0.8	6.6^a^	0.9	− 0.90	33
Palo Alto (2006)	9	6.0	1.7	5.4^a^	1.5	1.89	8
Berkeley 1-day (2006)	6	6.0	1.7	6.2	1.6	− 0.35	5
Total	122	6.3	1.1	6.5	1.2	− 1.58	121

*p < .05; **p < .01; ***p < .001

^a^Posttest scores significantly different from Palo Alto

^b^Posttest scores significantly different from Vallombrosa (Jan 2006)

An analysis of variance (ANOVA) confirmed that there were no pre-existing ego development differences at baseline among the participants between the sites, *F*(8, 9) = 0.38, *p* = .93. The pretest and posttest ego development scores were significantly correlated (*r* = .48, *p* < .001), whereby participants with higher pre-training scores also had higher post-training scores.

Because there are more older than younger adults in this sample, an analysis was conducted to test the relationship between age group and training effect. In total, 40 participants (33%, *n* = 40/122) increased one or more stages following the training. Proportionally, more 50–59 year olds (45%, *n* = 24/53) than the other age groups increased one or more stages after the training (20%, *n* = 1/5, under 30; 0%, *n* = 0/3, 30–39 year olds; 25%, *n* = 7/28, 40–49 year olds; and 24%, *n* = 8/33, 60+ year olds). To examine whether this trend was statistically significant, a *χ*^2^ test of independence was calculated by comparing age group and whether a participant experienced ego stage advancement of one or more stages following the intervention (or not). The *χ*^2^ test revealed no relationship between age and ego advancement.

An analysis was also conducted to test the relationship between participant gender and training effect. Although proportionally, more women (36%, *n* = 36/100) than men (18%, *n* = 4/22) advanced one or more stages following the training, a *χ*^2^ test of independence revealed no relationship between gender and ego advancement.

Two repeated-measures ANOVAs were conducted to test for within-subject ego development changes across the training sites. The first model examined the interaction term of ego development by training site type as a dichotomous variable (intensive site vs. introductory site); the model was not significant at the *p* < .05 level, *F*(1,120) = 1.32, *p* = .25. The second model examined the interaction term of ego development by individual training site; the model was also not significant at the *p* < .05 level, *F*(7, 114) = 1.40, *p* = .21.

Paired sample *t* tests were conducted to examine whether there were significant changes in ego development scores before compared to after the Enneagram training interventions at each site. Data revealed that the Asheville 2005 participant group showed a significant increase in ego development scores following the intervention, *t*(22) = − 3.36, *p* < .01. Table 4 summarizes the pre- and post-training ego development scores and the paired *t* test results.

In addition, an ANOVA of post-training scores by training site was conducted to examine whether the mean ego development scores differed between the sites after the Enneagram training. The ANOVA revealed an overall between-group difference of mean ego development score by training site post-training, *F*(7, 8) = 2.60, *p* < .05. Post hoc LSD tests revealed that at the *p* < .05 level, one introductory course in Palo Alto had significantly lower post-training scores than each of the Enneagram Intensive training sites, with the exception of Vallombrosa (January, 2006) (Table 4). In addition, the Intensive training site Asheville (2005) revealed a significantly higher post-training score than the Vallombrosa (Jan 2006) Intensive training site.

## Discussion

The aim of this study was to determine whether Enneagram training could: (a) promote ego development in adulthood; and (b) promote ego development to postconventional stages. It was proposed that the Enneagram personality system is uniquely positioned to promote adult ego development given its integrative framework that both imparts type-specific content tailored to the particular developmental trajectories of each type, and facilitates a process containing integral components to advancing developmental stages. The training program was evaluated by administering the WUSCT updated by Cook-Greuter and comparing participants receiving different durations of training to determine whether there was significantly greater stage transition for those participants with more opportunity to engage in learning the Enneagram system. Quasi-experimental design was used with repeated measures investigating whether individuals showed an increase in levels of ego development at 18 months follow-up.

The results partially affirmed our hypotheses and may be summarized as follows:

1. The Enneagram Intensive Training group, Asheville 2005, was the only training program that showed a significant increase in ego development scores between pretest and posttest.
2. The Enneagram Introductory Training group in Palo Alto showed significantly lower ego development scores at posttest than each of the Enneagram Intensive Training groups.

These findings will be discussed in terms of what we as the authors believe is unique to the Enneagram system in facilitating ego development in adulthood, explore the possible reasons for inconsistencies seen in the data, and future directions.

### Advancing Stages

A central question addressed by this study is whether advancing stages of ego development can be promoted through interventions in adulthood. The results confirm what research has begun to illuminate, namely that continued ego development is possible and may be facilitated through interventions (Chandler et al. 2005; Manners et al. 2004). We hypothesized that the Enneagram training would promote stage advancement and that the participants who received more extensive training would demonstrate greater advancement in developmental level. The results confirmed the hypothesis. While the 15–20 and 40–50 h training groups started at the same pretest levels of ego development, only the 40–50 h training group showed a statistically significant mean increase in stage of ego development (*p* < .05).

What naturally follows is the question: what about the intervention made it successful? From a content perspective, the Enneagram lays a strong theoretical foundation for how adults may progress and grow. Many integral components of lifespan development are incorporated into the system including motivation, mental schemata, personality structure, and temperament. These components are strongly knitted together in a manner that provides a taxonomy of traits and unique developmental trajectories across the lifespan. From a process perspective, the training integrates important components for promotion of ego development: an opportunity for participants to encounter the limitations of their worldview in a personally salient way while providing new perspectives (Manners et al. 2004; Saracino 2013).

Both the content and the process of the Enneagram training in this study were likely important for the promotion of growth. The Enneagram’s developmental roadmap for each type’s unique personality progression provides a sufficiently complex model to enable individuals to navigate their internal experience effectively. The integrative nature of this system is an ideal fit for a model because it includes the tools to effectively promote ego development in adulthood. The specific tools of process that facilitate ego development include: developing self-observation, empathy development, understanding of motivation and underlying beliefs, and what methods support further development.

Our study contained many variables known to improve emotional regulation and well-being, such as psycho-education (Mennin and Fresco 2009), mindfulness (Goldin and Gross 2010), and bodywork practices (Mehling et al. 2011). These factors are often thought of as being fertile ground for growth and advancing in levels of ego development. In addition, as described by Saracino (2013), it is possible that a key component of advancing ego development is the use of “type panels” as described earlier in this paper. Guided by a trained facilitator in a supportive and safe environment, panels of representatives of each type describe their automatic patterns of thinking, feeling, and behaving. Both speaker and audience are impacted by the candid sharing and vulnerability, as well as by recognition of the internal intelligence and consistency within each of the type patterns (Saracino 2013).

We believe that the panel method of the Narrative Tradition may significantly contribute to participant growth. Since this study did not assess these effects of the training experience, future research should replicate this study and test for the effectiveness of type panels in advancing ego development.

### Postconventional Development

According to Cook-Greuter (2002), only 7–17% of adults reach postconventional stages of development in adulthood. Our initial sample had a high percentage of participants already at the postconventional level, likely reflecting the particular characteristics of those interested in this training. The distribution of stages was similar to the normal population. However, for the participants in this study, after 18 months follow-up, approximately half of the participants were at a postconventional level of development, which is markedly different from what is considered a normal population distribution. The only other study to date reporting this level of postconventional development was a longitudinal study of the impact of TM (Chandler et al. 2005).

This finding represents the first empirical demonstration that rapid and significant stage advancement is possible. At 18 months follow-up, nearly one-third of participants advanced one or more levels. Advancing more than one level in an 18-month period is noteworthy given that rate of development in adulthood is generally viewed as slow if not stagnant (Wilber 2000). Cook-Greuter has extensive experience with the instrument and has scored over 14,000 protocols. After reviewing the data, Cook-Greuter noted it was surprising to see the number of participants who had advanced two or more levels of ego development between pretest and posttest (personal communication, July, 1 2010*)*. Given the crises we face in the modern day and the benefits to both the individual and society of individuals operating at postconventional stages, it is promising to see that there is possibility for intentional development in adulthood.

Additionally, there was an 84% return rate of the mailed Washington Sentence Completion Test. This high response rate likely indicates that the training was meaningful to the participants who enrolled in our courses. Furthermore, the high compliance rate also suggests that participants had a desire to contribute to the research of the Enneagram.

### Inconsistent Patterns in the Findings

The results showed that there were inconsistencies in the findings. The Minnesota Introductory group performed not as hypothesized, but instead demonstrated similar results to the Intensive groups with more training at posttest, all except for the Vallombrosa 2006 group. Additionally, the Palo Alto Introductory group had a lower posttest score than each of the Intensive groups. The Berkeley Introductory group did not show the same pattern. One reason may be the low number of participants in the Berkeley group, in which an effect size may not have indicated a statistical significance.

A closer look at the Introductory group will show that the hours varied between the three trainings. The Minnesota Introductory group was a weekend format of 13 h, the Palo Alto training was a 20-h training spread across 10 weeks, and the Berkeley training was a day-long training of approximately 6 h. The large variation between all three Introductory groups is likely a reason for the inconsistencies seen in the results. In contrast, the Intensive training groups consistently adhered to 45–50 h. However, the training sites all varied in location and time, which may also explain why there was not a statistical significance amongst the training groups. It is recommended that future studies control for the same number of hours as well as location and time frame between comparison or control groups.

Another limitation is that within all of the Enneagram training groups, there was a lack of control for monitoring participants that may have enrolled in additional Enneagram training programs. Participants who enrolled in additional trainings may have interfered as a confounding variable to the control. In future studies, it will be important to monitor stricter criteria in participants to account for this. For example, 14 participants who originally participated in the Introductory Minnesota group also enrolled in an Enneagram weekend personal development training before the 18-month posttest follow-up had been conducted. These 14 participants were thus excluded from inclusion in this study.

All Enneagram trainings in this study were taught by certified Enneagram teachers in the Narrative Tradition; however, the Enneagram Intensive training groups were all taught by core faculty of the Enneagram Narrative Tradition, compared to the Palo Alto course that was not taught by core faculty. It is recommended that future studies control for the differences in Enneagram instructors. Specifically, detailed post-training participant evaluations of the instructors and the trainings may yield more information about how these variables affected the outcome of ego development across training groups. Lastly, the cause of the low posttest score for the Enneagram Intensive training Vallombrosa January 2006 group is unknown. Recommendations made in this section may yield more consistent results that were not controlled for in this study.

### Additional Limitations

Limitations of the study also include the quasi-experimental design and sampling strategy. The self-selected sample may differ from the general population in regard to levels of motivation and psychological mindedness. However, while the participants were self-selected, this also enabled greater generalizability to real-world settings where most participants have sought out the training and are likely motivated. It is an important observation that many of these individuals seeking personal development were able to effectively achieve ego development and to unusual degrees of postconventional stages.

Another limitation is the predominantly female representation of 80% in the final sample of this study. Therefore the results cannot be generalized to the experience of males enrolled in the Enneagram trainings. We recommend that future research investigate the effects of ego development that is representative of males enrolled in Enneagram trainings.

### Future Research

The study achieved its aim of laying a foundation for the empirical investigation of the application of the Enneagram to facilitate development in adulthood. It demonstrated the promise of the Enneagram as a field of inquiry. We stress the importance of replicating this study in a more controlled experimental design to verify the findings presented here. Future empirical research that examines the contribution of both the content and process of the training is important to provide confirmation of the findings and feedback to facilitate improvements in the trainings.

## Conclusion

The Enneagram has been rapidly expanding in both psychotherapy and organizational development. However, the proliferative nature of its application is not mirrored in the sphere of psychological research and discourse (Bland 2010; Fitzsimons and Killen 2013). The findings from this intervention study provide important insights into the potential efficacy of the Enneagram system to promote ego development in adulthood and facilitate the achievement of postconventional growth.

Given the promising results of this study and widespread use of the system, the time is ripe for further empirical research. Like most theories, the Enneagram’s origins lie in the realm of assimilated wisdom and personal experiences. However, it would be beneficial for the Enneagram community to expand upon the current paucity of research and more fully enter the realm of scientific inquiry. An expansion of empirical research can provide a framework for meaningful discourse across disciplines, provoking new and interesting questions, deepening theory, and enriching collective knowledge. Finally, empirical research can strengthen and facilitate effective application of the system and further determine its validity, efficacy, best applications, ethical use, and impact on ego development.
